# First person – Xin Huang

**DOI:** 10.1242/bio.058547

**Published:** 2021-02-09

## Abstract

First Person is a series of interviews with the first authors of a selection of papers published in Biology Open, helping early-career researchers promote themselves alongside their papers. Xin Huang is first author on ‘Dissecting dynamics and differences of selective pressures in the evolution of human pigmentation’, published in BiO. Xin conducted the research described in this article while a PhD student in Li Jin, Yungang He's laboratory, Shanghai, China. He is now a Postdoctoral Research Associate in the lab of Ryan Gutenkunst at University of Arizona, Tucson, AZ, USA, investigating new methods and tools to quantify natural selection from large-scale population genomic data.


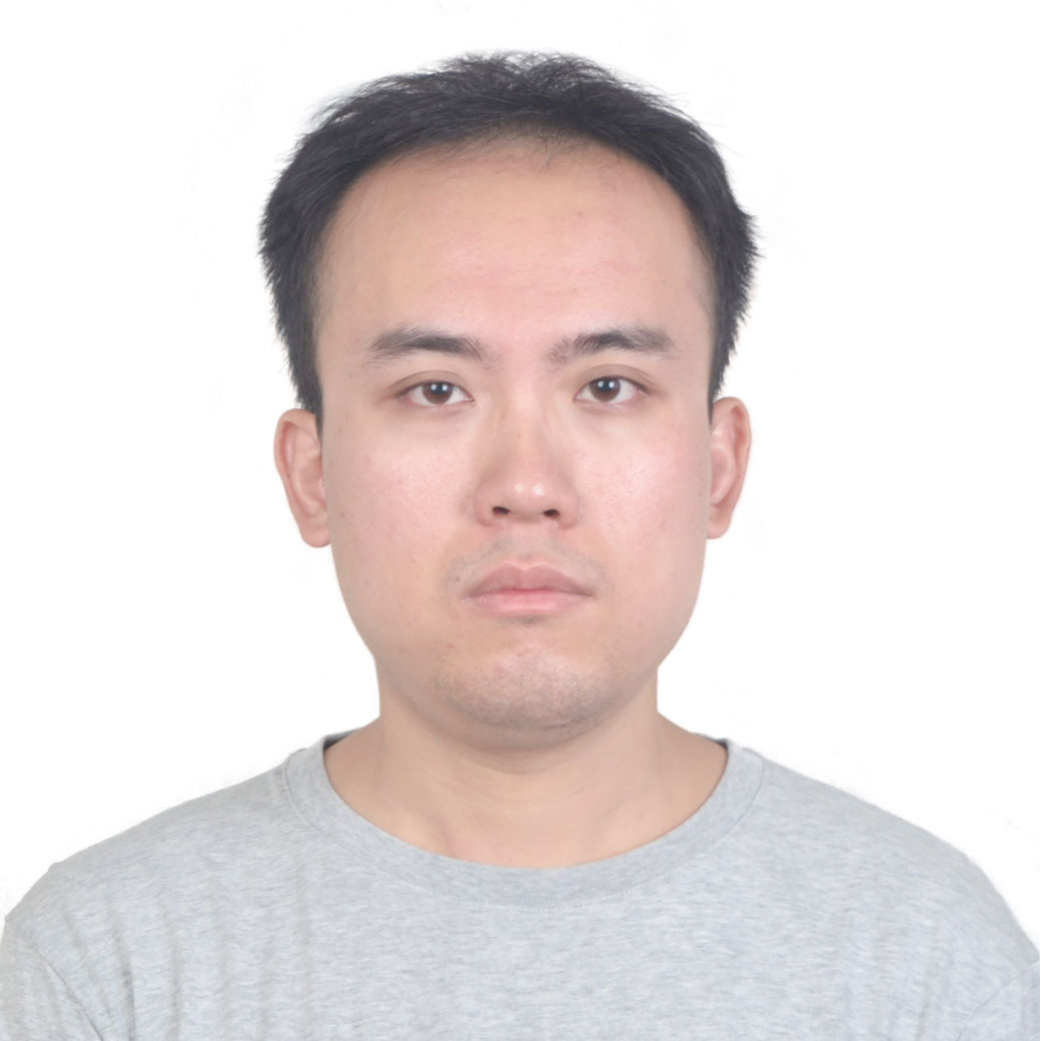


**Xin Huang**

**What is your scientific background and the general focus of your lab?**

I received my PhD training for population genetics in Shanghai Institutes for Biological Sciences. During my PhD study, I wrote a computer program called SeleDiff (Huang et al., 2019) to estimate selection coefficient differences between populations based on a previous study from our lab (He et al., 2015). I extended these previous works and conducted this study to explore the dynamics of natural selection in the evolution of human pigmentation.

Currently, I am a postdoctoral researcher in the lab of Dr Ryan Gutenkunst at the University of Arizona. Similar with my PhD study, my focus is to develop novel methods and apply them for quantifying natural selection from population genomic data.

**How would you explain the main findings of your paper to non-scientific family and friends?**

In this study, I developed a new method to estimate the strength of natural selection at different time periods. Then I applied this method to study the evolution of human pigmentation.

It is an important and interesting question to study how the strength of natural selection changes with time. The strength of natural selection may change through time due to various reasons, for example, the environmental change. However, most methods assume the strength of natural selection remains constant through time, only a few methods are available now for estimating how the strength of natural selection varies with time.

Human pigmentation refers to the colour of human skin, hair, and eye. It is one of the most diverse traits among human populations. It is also one of the traits that are probably under strong natural selection in humans. Therefore, human pigmentation is an ideal trait to be studied with my new method.

“I developed a new method to estimate the strength of natural selection at different time periods.”

**What are the potential implications of these results for your field of research?**

It is possible to develop new methods for quantifying the changes of natural selection through time. With more and more population genomic data available, such methods are compelling and would help researchers to study the dynamics of natural selection more comprehensively.

**What has surprised you the most while conducting your research?**

Although East Asians are populations with light pigmentation, the selective pressures on light pigmentation may reduce in East Asians recently.

**Figure:**
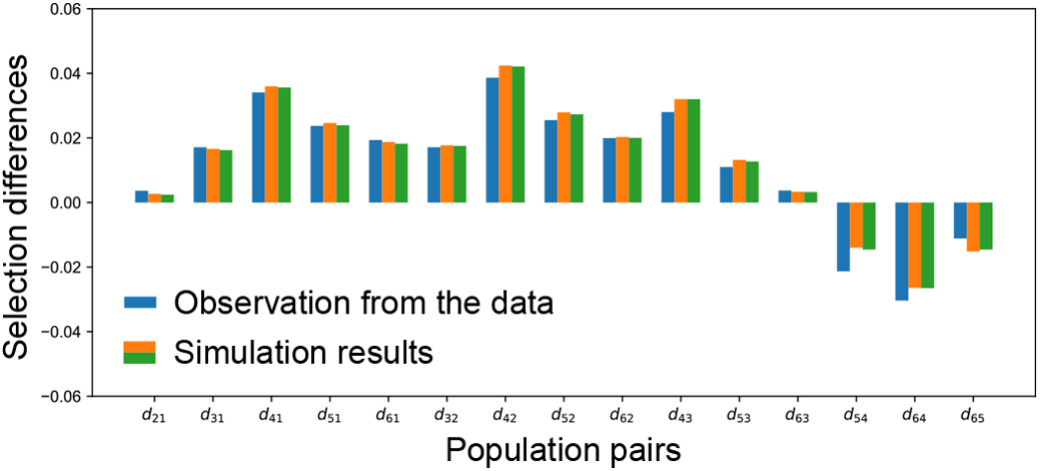
**Comparisons between simulation and observation.** The simulation results based on my new method matched the observed data well.

“Two decades ago, researchers only knew several mutations in a single gene named *MC1R* that may affect human pigmentation.”

**What, in your opinion, are some of the greatest achievements in your field and how has this influenced your research?**

I think high-throughput sequencing technology and genome-wide association studies are the greatest achievements. Two decades ago, researchers only knew several mutations in a single gene named *MC1R* that may affect human pigmentation. With high-throughput sequencing, thousands of genomes from different human populations became available. Using large-scale population genomic data and genome-wide association studies, more genes and variants were found to be associated with human pigmentation. Our understanding of the genetic architecture of human pigmentation as well as other complex traits in humans is becoming more and more comprehensive.

**What changes do you think could improve the professional lives of early-career scientists?**

I think our research community could provide more research grants to help early-career scientists. As the competition in academics becomes more and more intense, it is difficult for early-career scientists to seek research grants for supporting their positions. Therefore, I suggest our research community to give more grant opportunities for early-career scientists and consider more grant applications from early-career scientists.

**What's next for you?**

Currently, I am developing new methods for inferring the distribution of fitness effects from population genomic data. The distribution of fitness effects describes the proportions of deleterious, neutral, and beneficial new mutations across the genome. This is closely related to my PhD study and would help us to understand evolutionary theories or applications better.
